# Differential Expression of PACAP/VIP Receptors in the Post-Mortem CNS White Matter of Multiple Sclerosis Donors

**DOI:** 10.3390/ijms25168850

**Published:** 2024-08-14

**Authors:** Margo Iris Jansen, Giuseppe Musumeci, Alessandro Castorina

**Affiliations:** 1 Laboratory of Cellular & Molecular Neuroscience (LCMN), School of Life Sciences, Faculty of Science, University of Technology Sydney, P.O. Box 123, Sydney, NSW 2007, Australia; margo.jansen@student.uts.edu.au; 2 Department of Biomedical and Biotechnological Sciences, Human Anatomy and Histology Section, School of Medicine, University of Catania, 95123 Catania, Italy; g.musumeci@unict.it

**Keywords:** pituitary adenylate cyclase-activating polypeptide, vasoactive intestinal peptide, multiple sclerosis, normal-appearing white matter, relapsing-remitting MS, secondary progressive MS, primary progressive MS, demyelination

## Abstract

Pituitary adenylate cyclase-activating polypeptide (PACAP) and vasoactive intestinal peptide (VIP) are two neuroprotective and anti-inflammatory molecules of the central nervous system (CNS). Both bind to three G protein-coupled receptors, namely PAC1, VPAC1 and VPAC2, to elicit their beneficial effects in various CNS diseases, including multiple sclerosis (MS). In this study, we assessed the expression and distribution of PACAP/VIP receptors in the normal-appearing white matter (NAWM) of MS donors with a clinical history of either relapsing–remitting MS (RRMS), primary MS (PPMS), secondary progressive MS (SPMS) or in aged-matched non-MS controls. Gene expression studies revealed MS-subtype specific changes in PACAP and VIP and in the receptors’ levels in the NAWM, which were partly corroborated by immunohistochemical analyses. Most PAC1 immunoreactivity was restricted to myelin-producing cells, whereas VPAC1 reactivity was diffused within the neuropil and in axonal bundles, and VPAC2 in small vessel walls. Within and around lesioned areas, glial cells were the predominant populations showing reactivity for the different PACAP/VIP receptors, with distinctive patterns across MS subtypes. Together, these data identify the differential expression patterns of PACAP/VIP receptors among the different MS clinical entities. These results may offer opportunities for the development of personalized therapeutic approaches to treating MS and/or other demyelinating disorders.

## 1. Introduction

Multiple sclerosis (MS) is a chronic immune-mediated disease of the central nervous system (CNS) characterized by ongoing myelin loss, which leads to the formation of white matter lesions responsible for the subsequent neurodegeneration and functional impairment [1,2,3]. For yet undetermined reasons, the prevalence of MS is rising worldwide with an estimated 3 million people currently living with this diagnosis [4]. While the MS etiology remains partly unclear, genetic, environmental and lifestyle factors have been established as contributors to disease onset and development [5,6].

Historically, MS has been categorized into three main subtypes based on its clinical course, and although this classification is now becoming somewhat outdated, it is still in use in view of its validity for differentiating progressive forms of MS of unknown etiology from those secondary to milder cases at onset. Under this classification, the most common (and less aggressive) MS subtype is relapsing–remitting MS (RRMS), which accounts for approximately 87% of all MS cases [7]. RRMS is characterized by recurring exacerbations of symptoms (relapses) that are usually followed by some degree of recovery and the absence of clinical symptoms (remissions). In most cases, RRMS will progress into secondary progressive MS (SPMS), a stage in which patients will no longer experience episodes of recovery and the progressive worsening of symptoms becomes a prevalent clinical feature [8]. The third clinical subtype of MS is primary-progressive MS (PPMS). This form of MS is considered the most severe and is characterized by the relentless deterioration of symptoms after disease onset [9]. Currently, there is no cure for MS, and most disease-modifying therapies (DMTs) aim to tackle the dysfunctional immune system responsible for the myelin loss and/or attempt to ameliorate the large inflammatory component of the disease, especially in the earliest stages. However, despite the ever-growing arsenal of DMTs, not all MS patients respond well to currently available drugs, and many prefer symptomatic treatment over DMTs due to the severe adverse effects experienced with treatment [10]. This has prompted further studies regarding the identification of new therapeutic targets and the development of more effective treatment strategies.

A key pathological feature of MS is the chronic overactivation of the immune system against specific components of CNS myelin, which, combined with an inefficient reparative response by oligodendrocytes and their precursors, culminates in the formation of multi-focal scars/lesions, the typical hallmarks of an MS brain in diagnostic imaging [1]. In this context, the white matter that appears “normal” is referred to as normal-appearing white matter (NAWM). However, there is a growing body of evidence suggesting that, even in these seemingly unaffected areas, there may be subtle microscopic changes, such as axonal damage or alterations in cell density [11,12], which may not be easily detected by standard magnetic resonance imaging (MRI) techniques. In addition, there are reports suggesting that the NAWM of MS patients may present with subtle pathological and molecular signatures, such as signs of mild inflammation [13] and alterations in tight junctions [14,15,16], that are often difficult to capture using standard detection methods.

Vasoactive intestinal peptide (VIP) and pituitary adenylate-cyclase-activating peptide (PACAP) are two small neuropeptides produced and secreted by a multitude of CNS cells [17,18]. VIP and PACAP elicit their biological activities by binding to three G protein-coupled receptors (GPCRs) called VPAC1, VPAC2 and the PAC1 receptor. VIP, like PACAP, binds with high affinity to all three GPCRs; however, PACAP shows a higher affinity to PAC1 receptors than VIP (about 100-folds higher), making it a preferential PAC1 agonist [19,20]. Both VIP and PACAP are known for their anti-inflammatory and neuroprotective properties in the CNS and have been proposed as potential therapeutic targets in MS and associated disorders, such as optic neuritis [21,22]. Indeed, decreased serum VIP and PACAP levels have been detected in human blood and cerebrospinal fluid (CSF) samples, respectively [23,24], suggesting that these neuropeptides may also find application as biomarkers of disease and/or be utilized to monitor MS progression.

In the context of preclinical studies using experimental autoimmune encephalomyelitis (EAE), a well-established rodent model of MS, researchers have described divergent roles for some VIP/PACAP receptors and peptides. In fact, both PACAP- and VPAC1-deficient mice showed heightened resistance to EAE and reduced symptomatology, whereas VPAC2-deficient mice showed an exacerbated EAE pathology and more severe symptoms [25,26,27]. In addition, treatment with either PACAP or VIP ameliorated EAE severity, whereas unpublished data from our laboratory indicate that PACAP is most effective in preventing myelin loss in the cuprizone demyelination model. These data, despite being obtained from animal observations, pinpoint the potential distinctive beneficial roles of the two neuropeptides’ receptors with respect to different pathological domains of MS [28,29], and warrant further investigations on VIP/PACAP receptor expression and distribution in the human MS brain.

In the present study, we utilized a combination of post-mortem tissue sections (fixed) and case-matched fresh–frozen samples obtained from local brain tissue banks to explore the different expression levels and distribution of PAC1, VPAC1 and VPAC2 receptors in the NAWM of MS brains versus aged-matched non-MS controls. Furthermore, to define the pattern of changes in VIP/PACAP receptor levels across different MS clinical entities, analyses were also stratified based on the disease subtype. Finally, the study also investigated the distribution of VIP/PACAP receptors around and within lesioned areas of selected cases encompassing at least one chronically demyelinating lesion, unless otherwise stated.

## 2. Results

### 2.1. PACAP and VIP Gene Expression in the Normal-Appearing White Matter of Multiple Sclerosis Cases Reveals Subtype-Specific Changes

**Figure 1:**
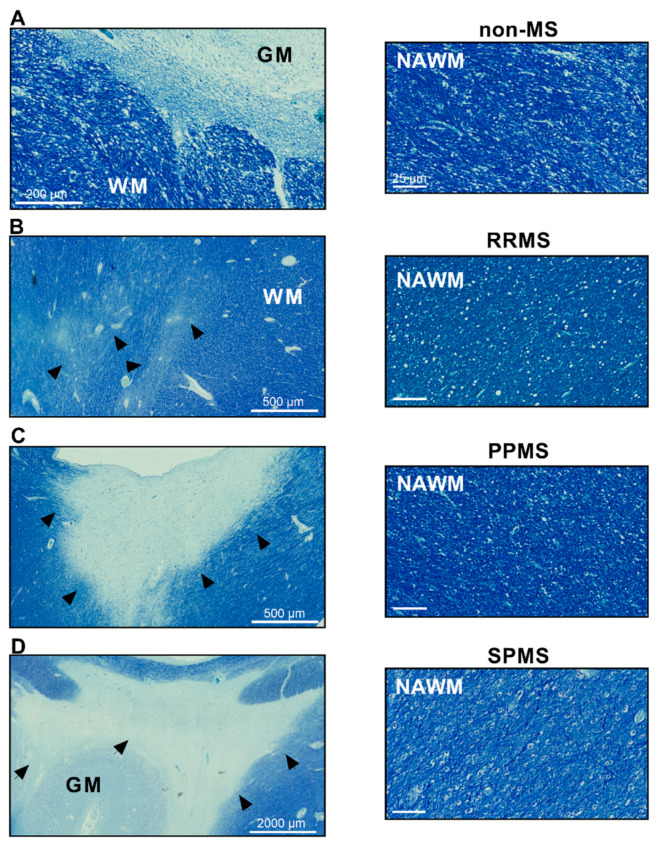
Representative lesions and normal-appearing white matter in human brain sections from donors with different MS subtypes. Luxol Fast Blue (LFB) staining shows the intense blue staining of myelinated fibers in the white matter (WM) of (**A**) non-MS donors, differentiating it from the less myelinated grey matter (GM). Evident discoloring of lesioned areas (indicated by black arrowheads) can be appreciated in sections from (**B**) RRMS, (**C**) PPMS and (**D**) SPMS cases. Myelin is stained blue, resulting in a clear distinction between GM and WM. Scale bar in (**A**) 200 µm, (**B**,**C**) 500 µm, (**D**) 2000 µm and NAWM (panels on the right) 25 µm. MS = multiple sclerosis, RRMS = relapsing–remitting MS, PPMS = primary-progressive MS, SPMS = secondary-progressive MS, GM = grey matter, WM = white matter.

Prior to examining the expression levels of the VIP/PACAP system in human normal-appearing white matter (NAWM), we conducted a preliminary evaluation of the NAWM integrity to identify gross structural alterations and locate lesioned areas within the tissue sections (Figure 1). To achieve this, we utilized Luxol Fast Blue (LFB) staining, a common histological technique that specifically stains myelin (in blue) and aids in the localization of lesions, which appear as discolored areas. This approach also enabled the definition of the regions of white and grey matter in the CNS adjacent to lesions, where present (WM and GM, respectively; Figure 1A and Figure 1D). As shown in the representative images (Figure 1B–D), at least one lesion was identified in each section representing the different MS clinical subtypes. No obvious differences in the LFB staining patterns were noticed in the NAWM amongst the selected cases.

**Figure 2:**
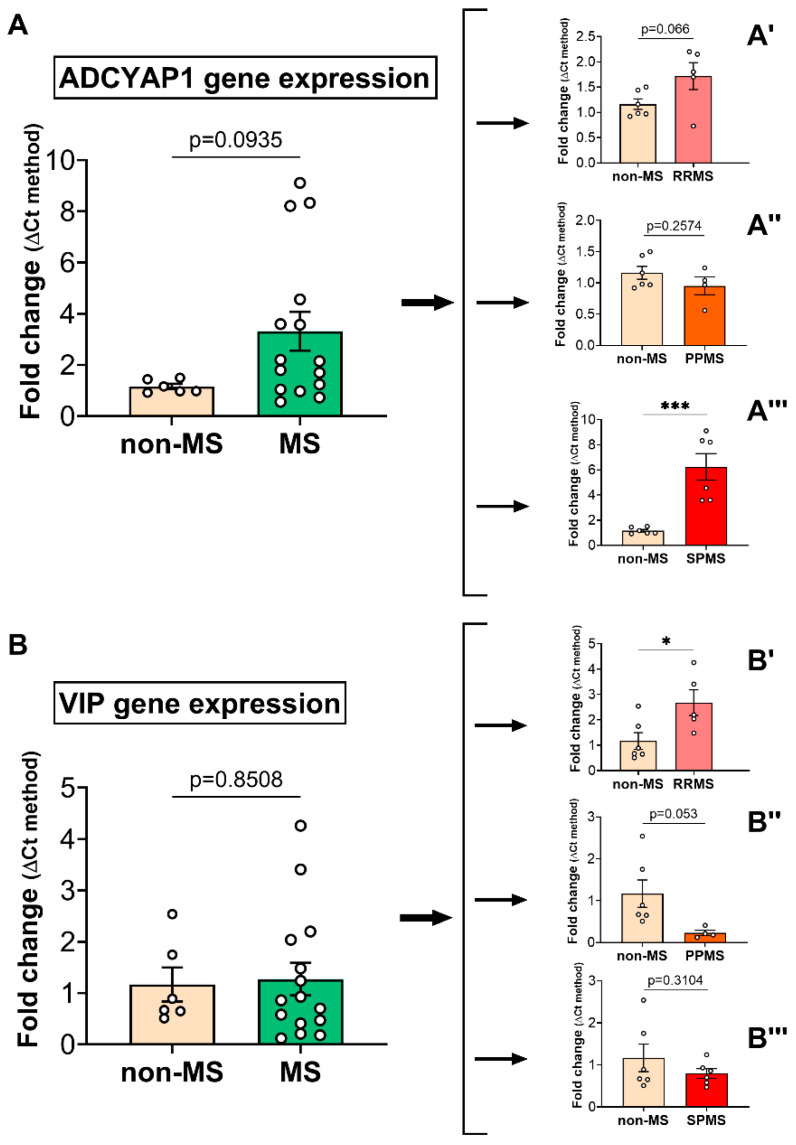
Differential expression of PACAP and VIP neuropeptide genes in the normal-appearing white matter of MS donors. (**A**) PACAP (gene name = ADCYAP1) expression was measured using RT-qPCR, comparing non-MS and MS cases. Further stratification of cases by clinical course, showing the expression levels of ADCYAP1 in (**A′**) non-MS vs. RRMS, (**A″**) non-MS vs. PPMS and (**A‴**) non-MS vs. SPMS. (**B**) VIP gene expression in non-MS vs. MS cases. A stratification similar to that in A demonstrates relative changes in the transcript levels between non-MS and (**B′**) RRMS, (**B″**) PPMS and (**B‴**) SPMS cases. The data shown are the mean fold change ± SEM, obtained from *n* = 6 (non-MS), *n* = 5 (RRMS), *n* = 6 (SPMS) and *n* = 4 (PPMS) cases. *p*-values > 0.05 are also shown. * *p* < 0.05 or *** *p* < 0.001 vs. non-MS, as determined by unpaired *t*-test. VIP = vasoactive intestinal peptide, PACAP = pituitary adenylate cyclase activating polypeptide, MS = multiple sclerosis, NAWM = normal-appearing white matter, RRMS = relapsing–remitting MS, PPMS = primary progressive MS, SPMS = secondary progressive MS.

Using real-time quantitative polymerase chain reaction (RT-qPCR), we measured the relative expression levels of the neuropeptides PACAP and VIP in the NAWM of MS patients and the non-MS control tissue samples (Figure 2). Upon the examining the PACAP (gene name *ADCYAP1*) and VIP expression in non-MS versus MS cases, no statistically significant differences were found (Figure 2A). However, the further stratification of data based on the clinical subtype demonstrated a significant increase in PACAP expression in SPMS cases (t_10_ = 4.790; *** *p* = 0.0007; Figure 2A‴) but not in RRMS and PPMS (*p* = 0.066 and *p* = 0.257, respectively; Figure 2A′ and Figure 2A″). VIP expression was significantly increased in RRMS (t_12_ = 2.454; * *p* < 0.05; Figure 2B′) and slightly downregulated in the NAWM of PPMS cases (t_12_ = 2.195; *p =* 0.0533; Figure 2B″). No statistically significant changes were observed in the NAWM of SPMS cases (t_10_ = 1.069; *p* = 0.3104; Figure 2B‴).

### 2.2. Differential Gene Expression Levels of the PACAP/VIP Receptors in the NAWM of MS Patients

**Figure 3:**
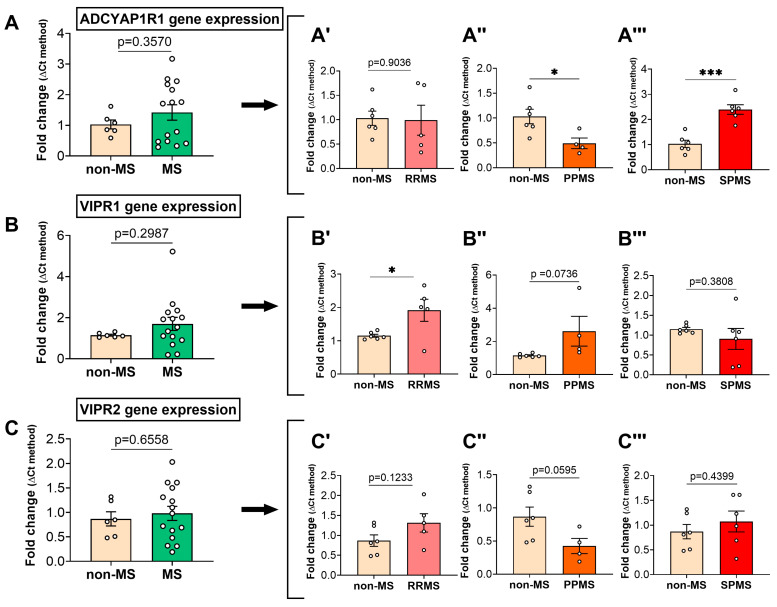
Differential expression of PAC1, VPAC1 and VPAC2 genes in the normal-appearing white matter of MS donors. Gene expression of (**A**) ADCYAP1R1 (aka PAC1), (**B**) VIPR1 (VPAC1) and (**C**) VIPR2 (VPAC2) in the NAWM of non-MS vs. MS donors. Upon stratification based on the clinical MS course, the gene expression levels of ADCYAP1R1, VIPR1 and VIPR2 were determined for (**A′**–**C′**) RRMS, (**A″**–**C″**) PPMS and (**A‴**–**C‴**) SPMS cases. The data shown are the mean fold change ± SEM, obtained from *n* = 6 (non-MS), *n* = 5 (RRMS), *n* = 6 (SPMS) and *n* = 4 (PPMS) cases. *p*-values > 0.05 are also shown. * *p* < 0.05 or *** *p* < 0.001 vs. non-MS, as determined by unpaired *t*-test. ADCYAP1R1 = Pituitary adenylate cyclase-activating polypeptide type I receptor, VIPR1 = Vasoactive intestinal polypeptide receptor 1, VIPR2 = Vasoactive intestinal polypeptide receptor 2, MS = multiple sclerosis, NAWM = normal-appearing white matter, RRMS = relapsing–remitting MS, PPMS = primary progressive MS, SPMS = secondary progressive MS.

Upon examining the gene expression levels of the PAC1 (*ADCYAP1R1*), VPAC1 (*VIPR1*) and VPAC2 (*VIPR2*) receptor genes in the NAWM of all MS patients combined, no statistically significant differences were observed (*p* = 0.357, *p* = 0.2987 and *p* = 0.6558, respectively; Figure 3A–C). However, comparisons of the MS cases based on the clinical course of the disease revealed subtype-specific differences in the expression of each PACAP/VIP receptor. Specifically, while *ADCYAP1R1* expression was still not significantly affected in the NAWM of RRMS cases vs. non-MS cases (t_9_ = 0.125; *p* = 0.9036; Figure 3A′), transcript levels were significantly downregulated in PPMS (t_8_ = 2.681; * *p* < 0.05; Figure 3A″). In contrast, they were remarkably increased in SPMS cases (t_10_ = 5.709; *** *p* < 0.001; Figure 3A‴).

*VIPR1* expression was significantly increased in the NAWM of RRMS cases (t_9_ = 2.527; * *p* < 0.05; Figure 3B′), as well as in PPMS cases, although not at a statistically significant level (t_8_ = 2.058; *p* = 0.074; Figure 3B‴). In contrast, no changes were identified when comparing the *VIPR1* expression in SPMS vs. non-MS cases (t_10_ = 0.917; *p* = 0.3801; Figure 3B″).

No significant changes were identified when analyses of the *VIPR2* gene expression were narrowed to RRMS cases (t_9_ = 1.7; *p* = 0.123; Figure 3C′). In contrast, gene expression was reduced in the NAWM of PPMS cases, bordering statistical significance (t_8_ = 2.195; *p* = 0.0595; Figure 3C″). No statistically significant changes were observed when analyzing the *VIPR2* gene expression in SPMS cases (t_10_ = 0.804; *p* = 0.44; Figure 3C‴).

### 2.3. Differential Expression and Distribution of PAC1 Receptors in the NAWM and Lesions of MS Donors

Further to our gene expression studies, we sought to determine if the observed changes in transcript levels were mirrored by comparable changes in PAC1 protein expression, and we also assessed cellular/tissue localization. For this purpose, we conducted immunohistochemistry in brain tissue sections containing NAWM in selected MS cases (RRMS, PPMS and SPMS), as well as in aged-matched non-MS controls.

**Figure 4:**
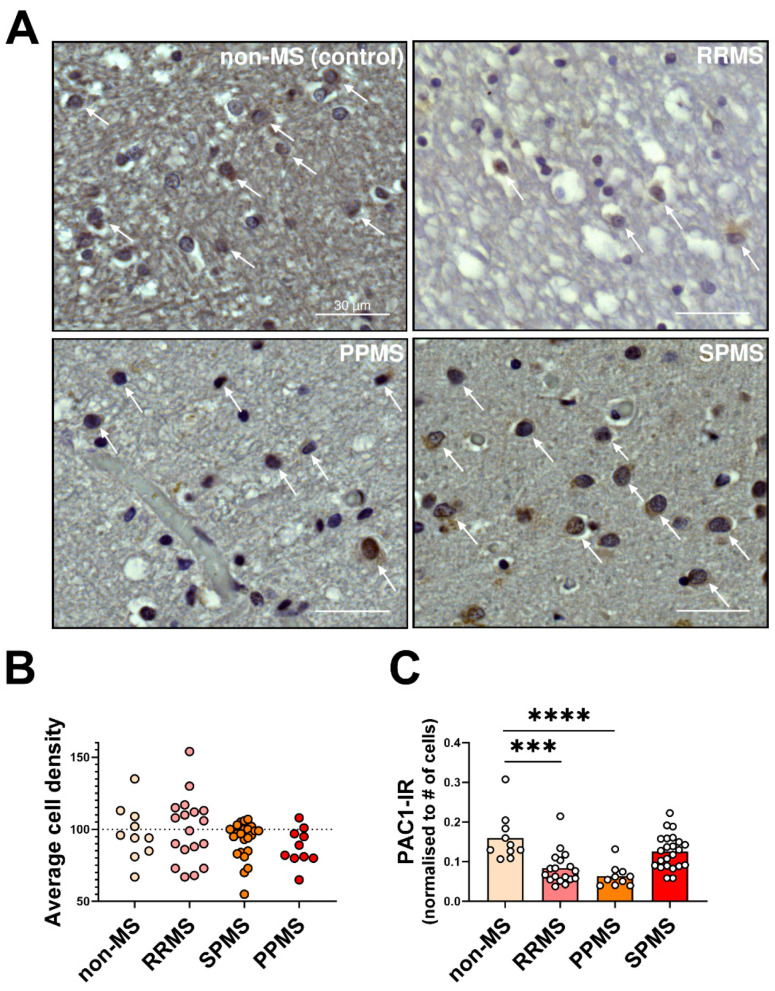
PAC1 immunoreactivity in the normal-appearing white matter of RRMS, PPMS and SPMS cases. (**A**) Representative images showing PAC1 immunoreactive cells in the NAWM of MS donors with a clinical history of RRMS, PPMS or SPMS and non-MS control cases. White arrows in each panel point to PAC1^+^ cells, which exhibit chromatin-dense and rounded/oval shaped nuclei, consistent with the oligodendrocyte/OPC morphology. (**B**) The average cell density (total # of cells per region of interest (ROI); ROI area = 1.23 mm^2^) was calculated using 2–4 ROIs from *n* = 5 (non-MS), *n* = 4 (PPMS), *n* = 6 (RRMS) and *n = 6* (SPMS) cases. (**C**) The PAC1 immunoreactivity in cells was determined by normalizing the mean PAC1 staining intensity/average # of cells counted within the same ROIs/cases as in (**B**). *** *p* < 0.001 or **** *p* < 0.0001 vs. non-MS cases, as determined by one-way ANOVA followed by Sidak’s post hoc test. Scale bar = 30 µm. OPC = Oligodendrocyte progenitor cell, PAC1 = Pituitary adenylate cyclase-activating polypeptide type I receptor, MS = multiple sclerosis, NAWM = normal-appearing white matter, RRMS = relapsing–remitting MS, PPMS = primary progressive MS, SPMS = secondary progressive MS.

As shown in the representative sections shown in Figure 4A, PAC1 immunoreactivity (IR) was distinctively segregated to the perinuclear and cytoplasmic compartments of cells that exhibited either euchromatic or heterochromatic nuclear patterns, typical histological features of oligodendrocytes and oligodendrocyte progenitor cells (OPCs), respectively. In addition, most of these PAC1^+^ cells demonstrated enlarged nuclei, especially in progressive MS cases (Figure 4A, left and right lower panels), an indication of underlying myelin pathology [30]. Stereological analyses determined that the average cell counts were not significantly different among MS subtypes (F_3,57_ = 1.3, *p* = 0.283; Figure 4B), suggesting that, at least in the NAWM, despite the signs of myelin pathology, myelin-producing cells are spared from any obvious cell loss.

In contrast to mRNA measurements, PAC1-IR (normalized by the number [#] of cells) was remarkably decreased in RRMS (F_3,57_ = 11.2, *** *p* < 0.001 vs. non-MS; Figure 4C). Instead, in PPMS cases, the PAC1 immunosignals correlated well with the transcript levels and were significantly reduced compared with non-MS cases (**** *p* < 0.0001); however, this was not seen in SPMS cases, as PAC1-IR was similar to non-MS controls (*p* = 0.121).

**Figure 5:**
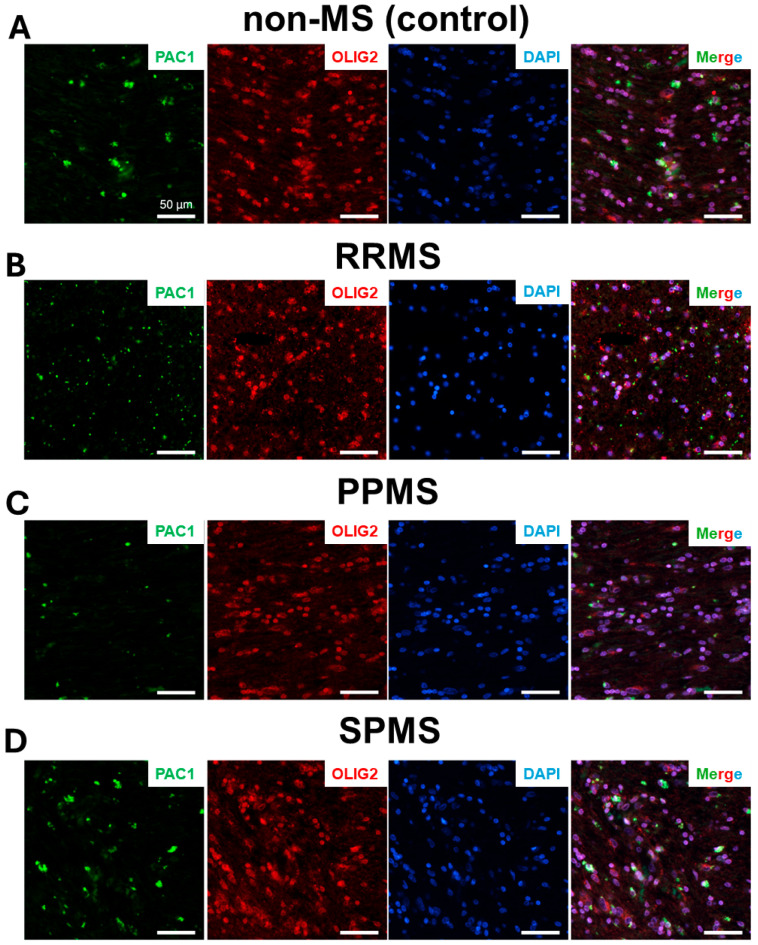
PAC1 co-localizes to OLIG2^+^ cells in the normal-appearing white matter of RRMS, PPMS and SPMS cases. Representative images showing PAC1 (green)/OLIG2 (red) colocalization in the NAWM of (**A**) non-MS, (**B**) RRMS, (**C**) PPMS or (**D**) SPMS donors. Nuclei were counterstained with DAPI (blue). Scale bar = 50 µm.

Additional co-immunolocalization experiments were conducted in selected RRMS, PPMS, SPMS and non-MS control cases to confirm whether PAC1 was mostly confined to cells belonging to the oligodendroglial lineage. As such, NAWM sections were co-stained with PAC1 and OLIG2 (Figure 5).

**Figure 6:**
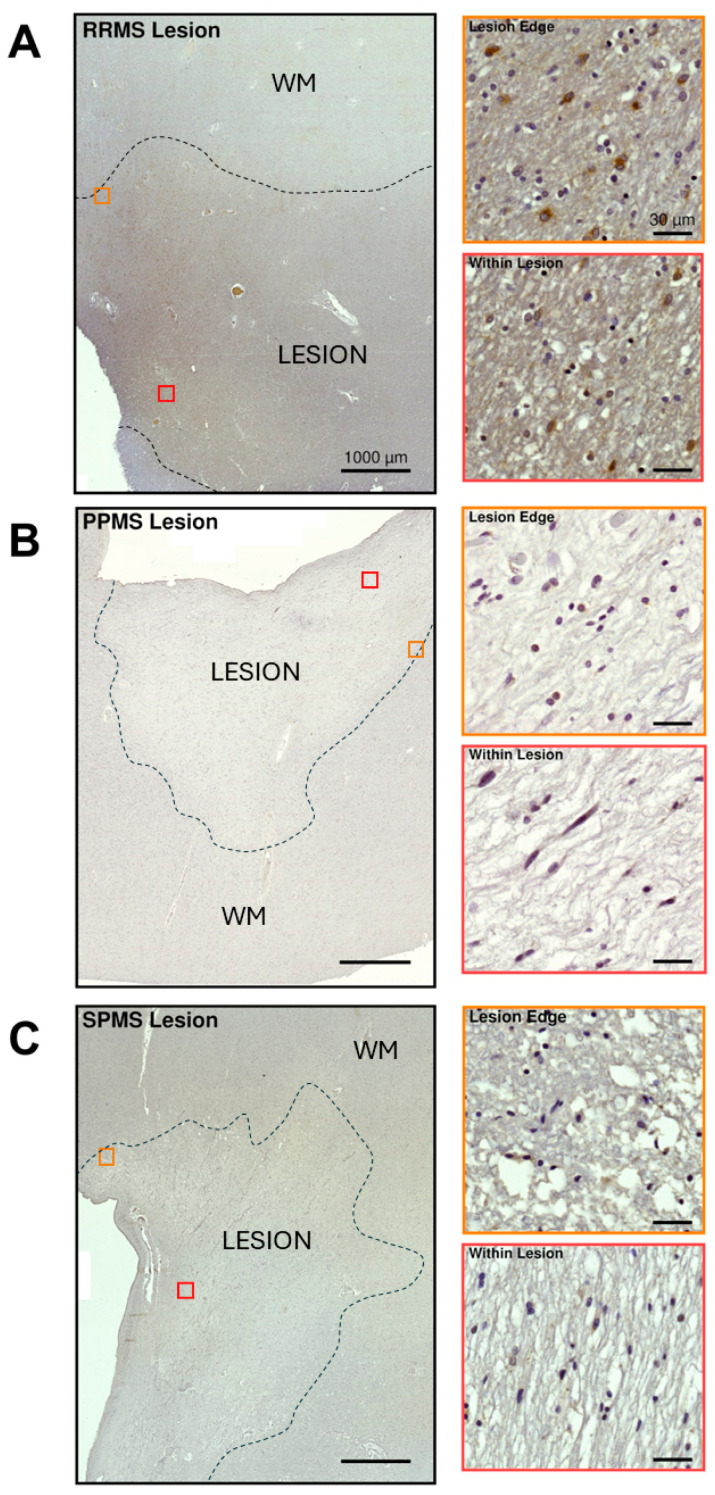
PAC1 immunoreactivity in representative white matter lesions from selected MS clinical cases. (**A**–**C**, left panels) Low-magnification images showing PAC1 immunoreactivity in a lesion taken from one RRMS, PPMS or SPMS-exemplary case. Lesion borders are demarcated by the black dashed lines. Scale bar = 1000 µm. (Insets in **A**–**C**) High-power images of ROIs in the left panels (orange and red squares) demonstrating PAC1^+^ staining around the lesion edge (top inset) and within the lesion (bottom inset) of the selected RRMS, PPMS and SPMS cases. Scale bar = 30 µm. WM = white matter.

To further characterize PAC1 expression and localization in MS lesions, we also conducted immunohistochemical analyses in lesioned areas in at least one representative MS case selected from each MS clinical subtype (Figure 6).

The first example shows a chronically active and moderately regenerating white matter lesion from an RRMS case (Figure 6A). Both intense and diffuse PAC1-IR was found within the lesion core and the peri-lesional areas, respectively (Figure 6A). When viewed at a higher magnification, strong PAC1 immunosignals were found along the lesion borders, which were mostly restricted to a subset of cells exhibiting hypochromatic, swollen nuclei and finely granular chromatin patterns, typical phenotypic features of gemistocytic (reactive) astrocytes [31]. In contrast, the majority of PAC1-IR was segregated to oligodendrocytes (i.e., cells with small dense nuclei surrounded by a clear halo) within the lesion center, although some PAC1^+^ astrocytes were still noticeable (insets in Figure 6A). Given the remarkable and well-defined PAC1 positivity seen in astrocyte-appearing cells found around the portrayed regenerating lesion in our exemplary RRMS case, we sought to conduct co-immunolocalization studies using the astrocytic marker glial fibrillary acidic protein (GFAP) and PAC1 to confirm cell specificity. Experiments confirmed that PAC1^+^ astrocytes were mostly abundant around the contours of the regenerating RRMS lesion rather than within the lesion core (Supplementary Figure S1).

Similar experiments were carried out in a selected PPMS lesion presenting overt histological signs of chronic demyelination (i.e., reduced cellularity and strong myelin loss). The lesion demonstrated a global downregulation of PAC1-IR and a well-defined area of discoloration (Figure 6B). The lesion edges displayed some weak PAC1 positivity, more frequently in cells with small, tubular-shaped nuclei (possibly microglia), as well as in scattered cells with nuclei showing the typical “clock-face” or “cartwheel” appearance of infiltrating plasma cells. Within the lesion, there were few PAC1^+^ cells exhibiting the morphological features of foamy macrophages and rare undefined cells with a spindle-like morphology, perhaps oligodendrocytes (insets in Figure 6B).

In the chronic demyelinating SPMS lesion, the distribution of PAC1-IR was similar to the PPMS case, with rather weak PAC1 immunosignals seen both within and around the lesioned area (Figure 6C). A few PAC1^+^ cells resembling microglia/macrophages were also found both around the edge and within the lesion, but there was no obvious evidence of PAC1^+^ oligodendrocytes, especially in the lesion center (insets in Figure 6C).

### 2.4. Differential Expression and Distribution of VPAC1 Receptors in the NAWM and Lesions of MS Donors

**Figure 7:**
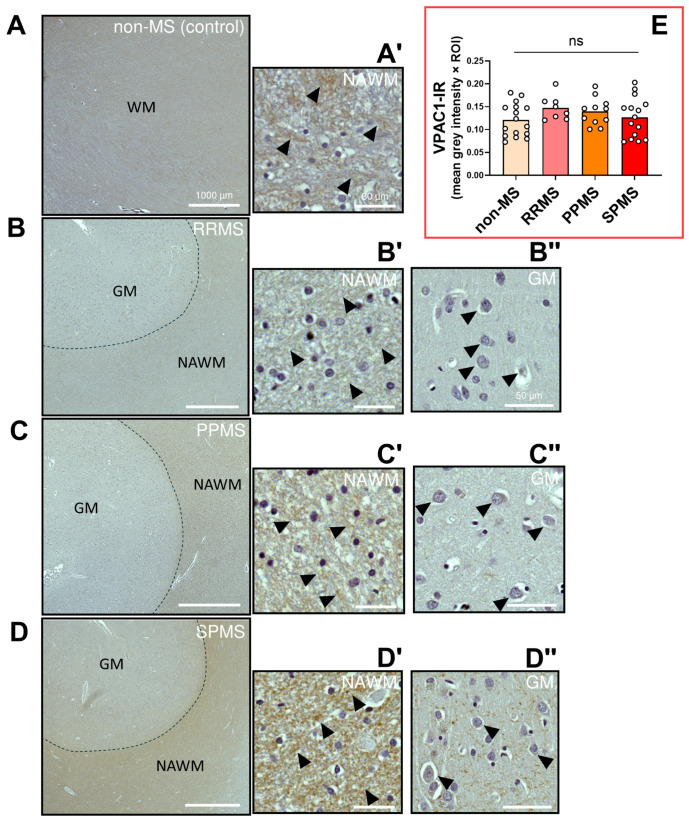
VPAC1 immunoreactivity in the normal-appearing white matter of RRMS, PPMS and SPMS cases. (**A**–**D**) Representative images depicting VPAC1 immunoreactive sites in the NAWM of MS donors with a clinical history of RRMS, PPMS or SPMS and non-MS control cases. Scale bar = 1000 µm. (**A′**–**D′**) Insets of the NAWM taken at a higher magnification. Black arrowheads point to VPAC1^+^ axonal fibers. Scale bar (NAWM) = 30 µm (**B″**–**D″**). Insets showing VPAC1^+^ in the grey matter of the selected cases. Black arrowheads indicate VPAC1^+^ neurons. Scale bar (GM) = 50 µm. (**E**) Bar graph showing the average VPAC1 immunoreactivity (IR) in the NAWM. The data shown are the mean grey intensity ± SEM and were calculated by averaging the grey intensity of 2–4 ROIs from *n* = 5 (non-MS), *n* = 4 (PPMS), *n* = 6 (RRMS) and *n* = 6 (SPMS) cases. Each ROI area = 1.23 mm^2^. No statistical significance was found using one-way ANOVA. Ns = Not significant. VPAC1 = Vasoactive Intestinal Peptide/Pituitary Adenylate Cyclase Activating Polypeptide Receptor 1, MS = multiple sclerosis, NAWM = normal-appearing white matter, WM = white matter, GM = grey matter, RRMS = relapsing–remitting MS, PPMS = primary progressive MS, SPMS = secondary progressive MS.

The immunohistochemistry performed to detect VPAC1 protein expression and distribution within the NAWM demonstrated that this PACAP/VIP receptor could not be localized to oligodendrocytes, its precursors, or other cell types, corroborating previous findings in the CNS white matter of rats and non-human primates [32,33]. Yet, moderate to strong VPAC1 immunosignals were observed in what appeared to be axonal bundles in RRMS, PPMS and SPMS cases (black arrowheads in Figure 7A′–D′). Notably, comparative analyses of VPAC1-IR did not reveal any significant differences amongst the different MS subtypes (F_3,46_ = 1.225, *p* = 0.3112; Figure 7E). Observations of VPAC1-IR within the adjacent grey matter (GM) of selected MS cases (black arrowheads in Figure 7B″–D″) showed moderate cytoplasmic reactivity in neurons and no staining of GM oligodendrocytes, providing some degree of assurance regarding the specificity of the antibody used for VPAC1 detection.

**Figure 8:**
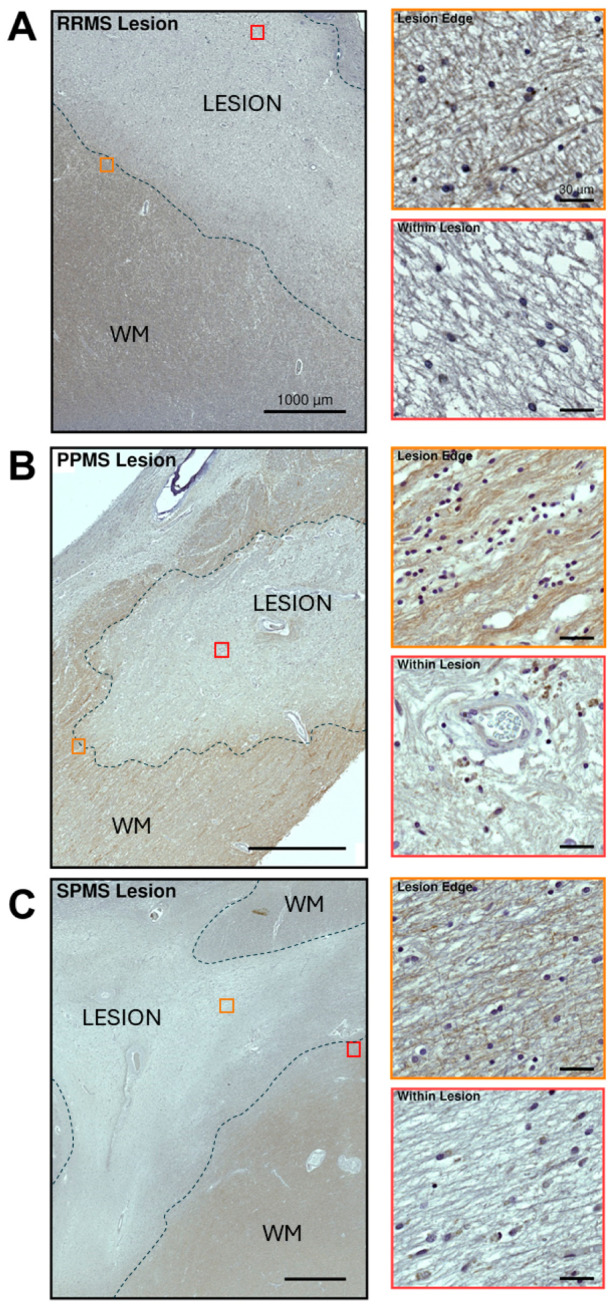
VPAC1 immunoreactivity in white matter lesions from selected MS clinical cases. (**A**–**C**, left panels) Low-magnification images showing VPAC1 immunoreactivity in a lesion taken from one RRMS, PPMS or SPMS-exemplary case. Lesion borders are demarcated by the black dashed lines. Scale bar = 1000 µm. (Insets in **A**–**C**) High-power images of ROIs in left panels (orange and red squares) demonstrating VPAC1^+^ staining around the lesion edge (top inset) and within the lesion (bottom inset) of the selected RRMS, PPMS and SPMS cases. Scale bar = 30 µm. WM = white matter.

Within and surrounding the lesions of our selected RRMS, PPMS or SPMS cases, VPAC1 protein was also distributed along axonal bundles, although VPAC1-IR was much weaker in damaged WM sites (Figure 8) than in the surrounding NAWM (Figure 7).

In our representative chronically active RRMS lesion, moderate to strong VPAC1 reactivity was localized in axons, but not in any glial, resident or infiltrating immune cells within or surrounding the lesion (Figure 8A). Interestingly, the insets of the lesion at a higher magnification demonstrated reduced VPAC1-IR along the lesion rim, with an almost complete discoloration (lack of IR) within the lesion core (insets in Figure 8A).

Immunohistochemical analyses of another chronic demyelinating lesion—although from a PPMS case—demonstrated moderate and axon-specific VPAC1-IR around the edges of the lesion, but less so within the lesion itself, where there was strong discoloration (Figure 8B). The lesion rim displayed mildly elevated cellularity, with several VPAC1^−^ cells exhibiting a small, rounded nucleus (presumably OPCs or oligodendrocytes) and a few weakly VPAC1^+^ cells with phenotypic features of microglia. As shown in the inset of the lesion core seen at a higher magnification, there were clusters of VPAC1^+^ microglial-like cells surrounding some intralesional small vessels, whereas the cells that configured as astrocytes were found in close apposition to the vessel walls and were all VPAC1^−^ (insets in Figure 8B).

The representative case shown in Figure 8C shows a typical chronic demyelinating lesion from a SPMS donor. The lesion presented little to no sign of regeneration. As in RRMS and PPMS cases, moderate VPAC1-IR was localized in axons passing through the white matter and adjacent to the lesion border, whereas IR was almost totally absent inside the lesion, with only a few glial cells displaying mild VPAC1 positivity (possibly microglia).

### 2.5. Differential Expression and Distribution of VPAC2 Receptors in the NAWM and Lesions of MS Donors

**Figure 9:**
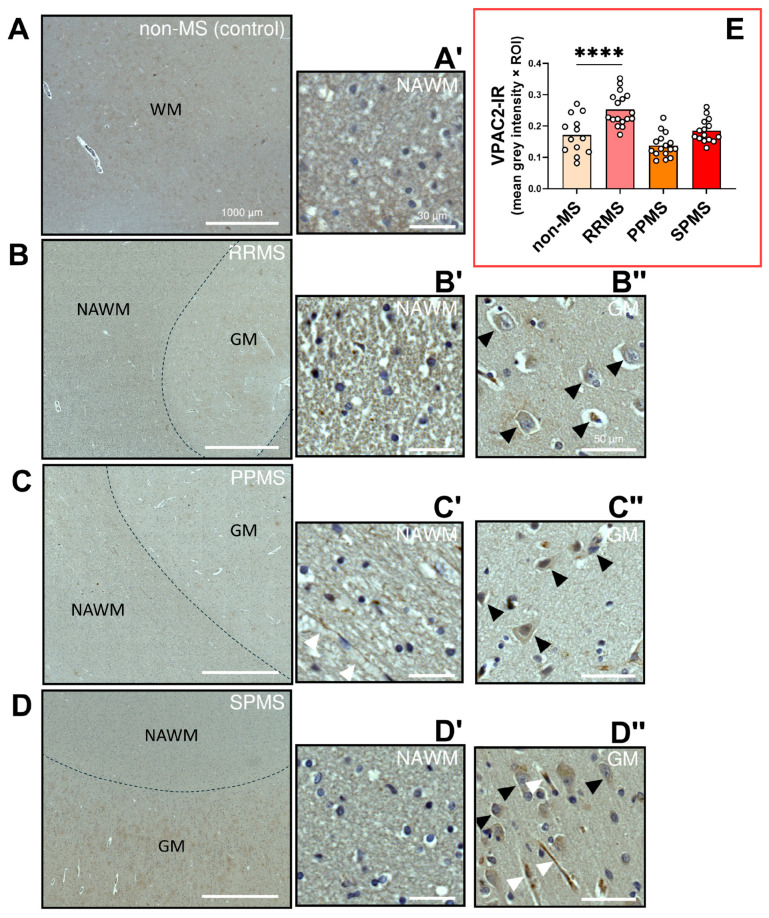
VPAC2 immunoreactivity in the normal-appearing white matter of RRMS, PPMS and SPMS cases. (**A**–**D**) Representative images depicting VPAC2 immunoreactive (IR) cells in the NAWM of MS donors with a clinical history of RRMS, PPMS or SPMS and non-MS controls. Scale bar = 1000 µm. (**A′**–**D′**) Insets of the NAWM taken at a higher magnification. White arrowheads in C′ show VPAC2^+^ vessel walls. Scale bar (NAWM) = 30 µm. (**B″**–**D″**) Insets showing VPAC2^+^ in the grey matter of the selected cases. Black arrowheads indicate VPAC2^+^ neurons, whereas white arrowheads show VPAC2-IR in axons. Scale bar (GM) = 50 µm. (**E**) Bar graph showing the average VPAC1 immunoreactivity (IR) in the NAWM. The data shown are the mean grey intensity ± SEM and were calculated by averaging the grey intensity of 2–4 ROIs from *n = 5* (non-MS), *n* = 4 (PPMS), *n* = 6 (RRMS) and *n* = 6 (SPMS) cases. Each ROI area = 1.23 mm^2^. **** *p* < 0.0001 vs. non-MS (control) cases, as determined by one-way ANOVA and Sidak’s post hoc test. VPAC2 = Vasoactive Intestinal Peptide/Pituitary Adenylate Cyclase Activating Polypeptide Receptor 1, MS = multiple sclerosis, NAWM = normal-appearing white matter, GM = grey matter, RRMS = relapsing–remitting MS, PPMS = primary progressive MS, SPMS = secondary progressive MS.

The immunohistochemistry for VPAC2 in brain tissue sections of donors with differing clinical disease courses (i.e., RRMS, PPMS or SPMS) did not reveal any cell-specific staining within the NAWM (Figure 9A). However, sparse and weak VPAC2-IR was identified in what appeared to be the walls of some small vessels infiltrating the NAWM (white arrowheads in Figure 9C′). Some mild/moderate cytoplasmic VPAC2 staining was detected in neurons (black arrowheads in Figure 9B″–D″), as well as in axons of the presented SPMS case (white arrowheads in Figure 9D″). Semi-quantitative analyses of VPAC2-IR identified a robust and statistically significant increase in VPAC2 staining in the NAWM of RRMS cases (F_3,57_ = 17.17, **** *p* < 0.0001; Figure 9E), but not in PPMS or SPMS cases.

**Figure 10:**
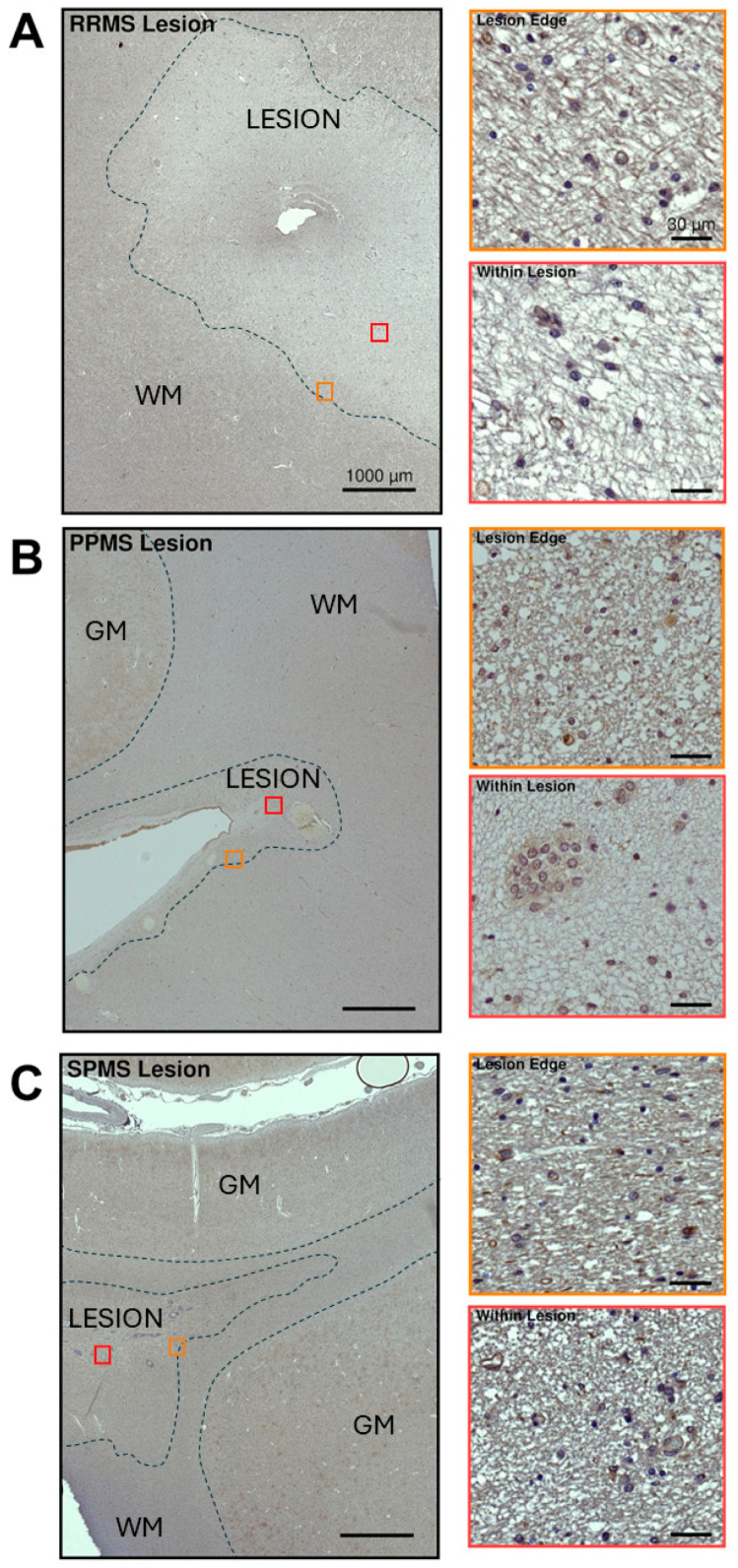
VPAC2 immunoreactivity in white matter lesions from selected MS clinical cases. (**A**–**C**, left panels) Low-magnification images showing VPAC2 immunoreactivity in a lesion taken from one RRMS, PPMS or SPMS-exemplary case. Lesion borders are demarcated by the black dashed lines. Scale bar = 1000 µm. (Insets in **A**–**C**) High-power images of ROIs in left panels (orange and red squared) demonstrating VPAC2 staining around the lesion edge (top inset) and within the lesion (bottom inset) of the selected RRMS, PPMS and SPMS cases. Scale bar = 30 µm. GM = grey matter; WM = white matter.

The immunohistochemical analysis of VPAC2-IR within and surrounding lesions demonstrated consistent staining in cells exhibiting a hypochromatic nucleus with astrocytic resemblance, specifically in representative RRMS and PPMS cases (Figure 10A,B).

In the reported RRMS case, depicting a chronically active and demyelinating lesion with minimal signs of regeneration, VPAC2^+^ cells displayed a scattered distribution both along the edges and within the lesion, where we also detected some isolated VPAC2^+^ cells with astroglial appearance (insets in Figure 10A).

In the chronically demyelinated PPMS case presented below, VPAC2^+^ cells exhibited the same histological features seen in RRMS cases, including the scattered distribution of IR astrocytes along the lesion edges (Figure 10B); however, the cell nuclei here appeared slightly smaller (top inset in Figure 10B), suggesting a non-reactive/resting phenotype. In contrast, VPAC2^+^ cells with swollen nuclei and organized in isolated clusters were found within the lesion core (bottom inset in Figure 10B).

Finally, in a chronically active SPMS lesion with moderate myelin loss and minimal evidence of remyelination (Figure 10C), focal VPAC2-IR was detected in isolated cells with microglial resemblance, both at the edge and within the center of the lesion (insets in Figure 10C).

## 3. Discussion

In this study, we aimed to provide a comprehensive overview of the changes in the expression and distribution of PACAP and VIP receptors in the CNS white matter of MS patients. To our knowledge, there are no studies portraying the differential expression levels of PACAP/VIP receptors in the human brain, let alone in the brain of MS patients. In fact, only a handful of investigations describing the expression of these neuropeptide receptors in the rodent and non-human primate brain have been published so far [32,33,34]. Therefore, this study provides novel and important findings on the differential expression of PACAP/VIP receptors in the NAWM and provides some insights from the analyses of representative lesions from donors with different clinical courses of MS.

After stratifying data according to the clinical disease progression, we identified several disruptions in the expression of PACAP/VIP receptors, some of which appeared to be dependent on the clinical progression of the disease. Indeed, both the PACAP and PAC1 gene expression levels were unaltered in the NAWM of RRMS patients; however, the quantification of immunosignals demonstrated that PAC1-IR was decreased in these same cases. Instead, in SPMS cases, PAC1 transcripts were increased in the NAWM, although this could not be confirmed at the protein level by immunolocalization studies. Only in PPMS cases was there complete congruence between gene and protein expression, as the PAC1 transcripts and immunoreactivity intensities were reduced in both cases.

PAC1 reactivity in the NAWM of MS patients was observed mainly in subpopulations of oligodendrocytes and OPCs. These discoveries were confirmed by co-immunolocalization studies demonstrating the presence of varying numbers of PAC1^+^/OLIG2^+^ cells within the NAWM across the different MS subtypes. This finding aligns with previous work performed in rats and non-human primates, further confirming that PAC1 is the only PACAP/VIP receptor to be expressed in the CNS white matter, and specifically in myelin-producing cells [32,33]. Indeed, in vitro data have shown the expression of functional PAC1 receptors in OPCs and mature oligodendrocytes [35]. Interestingly, the PAC1-preferring agonist PACAP exerts opposite effects in central vs. peripheral myelin cells. In OPCs, PACAP treatment delays cell maturation but stimulates proliferation, whilst in peripheral myelin cells (i.e., Schwann cells), it promotes cell differentiation and enhances the expression of myelin markers [35,36,37,38]. In the CNS, PACAP-mediated signalling is therefore thought to be crucial for the temporal control of myelin production [39]. As such, the reduced PAC1 expression in the NAWM of RRMS and SPMS cases might signify an increased demand for differentiation/myelination at the expense of OPC proliferation in chronically damaged white matter.

Around the lesion edges, PAC1 and VPAC2 reactivity was mostly seen in cells resembling microglia as well as infiltrating peripheral cells, including plasma cells. Whilst not a prevalent feature of early MS lesions, it is not uncommon to identify plasma cells infiltrates in chronic lesions of advanced MS cases [40]. However, at this stage, we are unable to determine the significance of PAC1 or other PACAP/VIP receptors in plasma cells, although we cannot rule out the possible involvement of the receptors in regulating antibody production and downstream inflammatory cascades. Preclinical data from EAE mice showed that the loss of PAC1 or VPAC2 aggravated symptoms in comparison to wild-type mice [25,27,41]. Since inflammation is a key component of MS pathology, our results highlight the potential importance of increased PAC1 and/or VPAC2 expression in reactive glial cells surrounding lesions [42,43]. Both PACAP and VIP have been hypothesized to stimulate phagocytosis in the CNS [44]. Since the effective phagocytosis of myelin debris around lesions is a crucial step in fostering a healthy microenvironment that promotes the maturation of OPCs and enhances remyelination [45,46], PAC1^+^ or VPAC2^+^ microglia in and surrounding lesions might have a role in regulating the phagocytosis of myelin debris [47] and/or the active regenerative processes driven by reparative astrocytes [48]. Both around and within the lesion of the RRMS case presented in this study, we also observed several PAC1^+^ cells with obvious histological features of reactive astrocytes; this was confirmed by co-immunofluorescence using the astrocyte marker glial fibrillary acidic protein (GFAP) (Figure S1). Reactive astrocytes in this example of a mildly active lesion might be there to promote repair; therefore, increased PAC1 expression in these cells might be of potential therapeutic significance [48,49]. Future studies addressing the importance of astrocyte-specific PAC1 receptor expression in remyelination may help shed more light on the role of these glial cells in myelin repair, especially in patients with progressive MS. Furthermore, our interpretations regarding the cell-specific expression of different PACAP/VIP receptors should be taken with caution, as our results were obtained from an individual RRMS case. Therefore, more comprehensive assessments of PACAP/VIP receptors at the cell-resolution level and in diverse lesion types (i.e., active, inactive, smoldering, shadow plaques, remyelinating lesions, etc.) are warranted to capture the full spectrum of changes needed to comprehend how these neuropeptide receptors contribute to glial cell functionality during lesion development or at different stages of recovery.

Our analyses of VPAC1 gene expression demonstrated robust increases in RRMS cases and only marginal changes in PPMS cases. Although the immunohistochemistry performed did not demonstrate significant increases in VPAC1 expression among the different MS subtypes, VPAC1 immunosignals were distributed in a diffuse pattern along the axonal bundles traversing the NAWM, and less than in any other local or infiltrating cells. At a glance, our VPAC1 staining resembled background staining; however, staining within the grey matter was neuron-specific, increasing our confidence regarding the specificity of the antibody.

Preclinical studies in VPAC1-deficient mice have shown reduced disease severity following EAE, suggesting that the receptor may play a role in regulating or perhaps sustaining the inflammatory response during experimental demyelination [26]. Therefore, it was surprising to observe an increase in VPAC1 expression in the NAWM of RRMS and PPMS cases, especially considering that, in these donors, most lesions were chronically demyelinating, with scarce evidence of inflammation. However, VPAC1 signaling is also linked to neuroprotection [50]; therefore, it cannot be excluded that the increased VPAC1 expression in the reported MS cases reflects the homeostatic neuroprotective response of stressed axons trying to retain functionality in a non-physiological CNS microenvironment, such as in the NAWM of MS patients.

With regard to VPAC2 expression, we did not find significant changes in gene expression amongst the different MS cases; however, there were trends towards increased mRNA expression in RRMS cases that were corroborated by robust increases in VPAC2-IR. In contrast, the VPAC2 gene and IR were reduced in PPMS cases. VPAC2 is normally upregulated under neuroinflammatory conditions [51], as it is generally considered an inflammatory sensor of the CNS [52]. From an immunohistochemical standpoint, VPAC2-IR was mostly expressed in endothelial cells of small infiltrating vessels present in the NAWM of PPMS cases. The expression of VPAC2 in infiltrating vessels might be the result of a subtle microvascular pathology or may represent a physiological adjustment of the vascular compartment to preserve blood–brain barrier (BBB) maintenance, since VIP treatment has previously been shown to reduce BBB permeability [53]. However, as we did not find similar staining patterns in other MS subtypes, including PPMS cases, future research is warranted to determine the role of the VIP/VPAC2 axis in BBB homeostasis.

It is important to address a few limitations of the current study. Firstly, our cell identification was based on the morphological appearance and certain histological features of CNS cells. Whilst this approach may not be completely accurate and can be prone to interpretation biases, it remains an acceptable solution when performing histopathological evaluations of CNS tissues. However, a way to circumvent these limitations would have been to conduct experiments following a more integrated approach involving the co-staining of sections with specific glial or immune cell markers (which were conducted in certain instances; please see Figure 5 and Figure S1) concurrent with other investigative modalities (i.e., Western blot and other molecular diagnostic tools). However, given the limited tissue availability, we based most of the identification of cell types on prototypical morphological features. It should also be noted that the main goal of this study was to assess the expression and distribution of PACAP/VIP receptors in the NAWM of donors with different clinical subtypes of MS, whereas our immunohistochemical studies in exemplary lesions were solely conducted with the purpose of providing some viable examples of how PACAP/VIP receptor distribution would vary across cell types in and surrounding damaged WM. In this regard, most of the assessed lesions were chronically active demyelinating lesions with minimal signs of regeneration (except for our exemplary RRMS case, which showed moderate signs of remyelination). Therefore, additional studies should address how these neuropeptides and related receptors are affected in non-remyelinating vs. remyelinating lesions; this would represent an important advancement in this specific field of research. Further limitations were related to the heterogeneity of the age of our cohort and staining across each subcategory of MS cases, which increased the intragroup variability when attempting semi-quantifications of IR and may have introduced a sampling bias. Further work using much larger cohorts with narrower age differences are needed to expand our current findings. Nonetheless, despite these technical challenges, we were glad that our PACAP/VIP receptor antibodies were rather specific, as demonstrated by the cytoplasmic staining of neurons in the GM. Additionally, the implementation of conditional knockout animal models, perhaps associated with a high-throughput single-cell RNA sequencing and/or in situ hybridization study, may represent a future goal of our research, as these studies will help establish the role of the VIP/PACAP receptors in individual cell types both in the NAWM and in MS lesions.

Overall, this study demonstrated a heterogenous pattern of expression and distribution in PACAP/VIP receptors in the NAWM of MS patients, which seemed to depend on the MS and receptor subtypes. In this regard, it is well established that relapsing and progressive MS cases display several pathological differences, including the degree and duration of inflammation, the regenerative activity and more [54,55]. These are likely to extend far beyond the lesion site [56] and it is not unreasonable to believe that these factors may cause subtle alterations in the NAWM, including the changes in VIP/PACAP receptors reported in this study. As such, this additional information could be useful to better understand how this neuropeptide system operates in the CNS of people with different clinical courses of MS.

## 4. Materials and Methods

### 4.1. Human Postmortem Brain Tissue

The MS and non-MS control tissue was supplied by the MS Research Australia Brain Bank (Tissue Transfer Deed—CT31920, approved on 21 June 2021) and the Victorian Brain Bank (Material Transfer Deed—VBB.19.07, approved on 16 January 2020). Snap-frozen tissue blocks (~100 mg each) were obtained and used for RNA isolation, cDNA synthesis and subsequent real-time qPCR analyses. For immunohistochemistry, fixed tissue sections cut at 5 μm and encompassing at least one lesion per case were provided for downstream analyses. Tissue sections were collected from a total of 6 individuals with RRMS, 6 with SPMS and 4 with a diagnosis of PPMS. Five age-matched cases from non-MS donors were included as controls in each experiment. A detailed overview of the demographic and clinical history of the donors is shown in Table 1.

### 4.2. Normal-Appearing White Matter Dissection and RNA Extraction

The RNA extraction of NAWM samples was performed as described previously [57]. Briefly, total RNA was extracted from micro-dissected snap-frozen WM shavings (each weighing about 120 mg) under RNase-free conditions using TRIreagent (Sigma-Aldrich, Castle Hill, NSW, Australia). Given that there is no universal agreed-upon distance to determine the diffusion of a lesion’s pathology, we empirically considered the NAWM in those tissue samples that were placed at a distance of >0.7 mm from the closest boundary of a lesion. After a tissue homogenization step, the samples were centrifuged (12,000× *g* at 4 °C) in the presence of 200 μL of chloroform (Sigma-Aldrich, Castle Hill, NSW, Australia). The RNA fraction was precipitated using 2-propanol (Sigma-Aldrich, Castle Hill, NSW, Australia) and spun down. The obtained RNA was treated with DNase I (Thermo Fisher Scientific, Scoresby, VIC, Australia), followed by a clean-up step using the RNeasy Micro Kit (Qiagen, Clayton, VIC, Australia). The RNA concentrations were determined using a NanoDrop™ 2000 spectrophotometer (Thermo Fisher Scientific, Scoresby, VIC, Australia).

### 4.3. Real-Time Quantitative Polymerase Chain Reaction (RT-qPCR)

Single-stranded cDNA was synthesized from the isolated total RNA using the Tetro cDNA synthesis kit (Bioline, Narellan, NSW, Australia), according to the manufacturer’s instructions. RT-qPCR was performed to analyze the mRNA levels of *ADCYAP1* (PACAP), *VIP*, *ADCYAP1R1* (PAC1), *VIPR1* (VPAC1) and *VIPR2* (VPAC2). Ribosomal protein S18 (*RPS18*) was used as a housekeeping gene. A detailed overview of the primer sequences can be found in Table 2. The RT-qPCR for each reaction was performed on CFX96 Real-Time System (C1000 Touch Thermal Cycler) using iTaq Universal SYBR Green Master Mix (Biorad, Australia). Each reaction contained a final concentration of 100 ng of cDNA, 400 nM of forward and reverse primer, and 5 µL of iTaq Universal SYBR Green Master Mix. The relative gene expression changes were calculated using the ∆∆Ct method, as described previously [58].

### 4.4. Immunohistochemistry

The tissue slides obtained from the Victorian Brain Bank and MS brain bank from non-MS and MS donor samples were deparaffinated in xylene and rehydrated through decreasing ratios of ethanol to water. A mild-heat antigen retrieval step (10 mM of sodium citrate, 0.05% Tween 20, pH 6.0; 15 min) was performed to unmask antigenic epitopes and improve antibody binding. The following antibodies were used for staining: Rb-αPAC1 (1:250, GeneTex, Irvine, CA, USA, Cat# GTX30026, RRID:AB_3097721), Rb-αVIPR1 (1:250, Sigma-Aldrich, Castle Hill, NSW, Australia, Cat#SAB4503084, RRID:AB_10751033), Rb-αVIPR2 (1:250, Millipore, Sigma-Aldrich, Castle Hill. NSW, Australia, Cat#AB2266, RRID:AB_10807709). Immunoreactivity was detected using the Rabbit-specific HRP/DAB (ABC) Detection IHC Kit (Abcam, VIC, Australia, Cat# ab64261, RRID:AB_2810213), according to the manufacturer’s instructions. Counterstaining using Hematoxylin (Lillie Mayer’s, Point of Care Diagnostics, North Rocks, NSW, Australia) was performed to visualize the cell nuclei. The slides were subsequently dehydrated with increasing concentrations of ethanol and xylene and mounted using VectaMount Express Mounting medium (H-5700-60, Abacus DX, Cannon Hill, QLD, Australia). Images were taken using the ZEISS AxioScan Z1 (Carl Zeiss Australasia, Macquarie Park, NSW, Australia) at ×20 magnification. Image analysis was performed using ImageJ (version 1.53k). Briefly, each image was deconvolved using the deconvolution function of ImageJ, by selecting the DAB deconvolution from the available options. This function splits the image into separate channels showing either DAB staining or hematoxylin counterstaining. Then, a threshold was applied to the DAB channel to remove any background staining. Next, 2–4 ROIs (area = 1.23 mm^2^) per case were randomly selected from the NAWM, added to the ROI manager, and saved for subsequent grey intensity measurements. Using the Analyze > Measure function, the intensities of each ROI were calculated and exported to a spreadsheet. For the calculation of the normalized staining intensity (based on # of cells), the hematoxylin channel was also used to determine the total number of nuclei per ROI. The mean grey intensity was then divided by the total number of cells. The intensity values shown in graphs refer to each ROI (pseudo-replicates) to better reflect the heterogeneity of staining within a given section/case. The exact number of cases (biological replicates) that were analyzed is reported in each corresponding Figure legend. Images were generated using OMERO.Figure (v4.2.0).

### 4.5. Immunofluorescence

Immunofluorescence staining was performed in 2–3 representative sections taken from non-MS, RRMS, PPMS and SPMS. Briefly, the NAWM sections were deparaffinized and rehydrated using xylene and decreasing ratios of ethanol to water. Following a mild antigen retrieval step (10 mM of citric acid, 0.05% Tween-20, pH 6.0; 15 min), an auto-fluorescence quenching step was performed using 0.25% NH_3_ in 70% ethanol for 1 h at RT. Slides were washed in PBS-T (0.05% Tween-20 in PBS) and permeabilized for 20 min (0.4% Triton-X100 in PBS). To block endogenous peroxidase activity, the sections were submerged in 3% hydrogen peroxide (3% H_2_O_2_ in methanol; 15 min), followed by a washing step. The sections were blocked for 1 h in 5% BSA (0.2% gelatin, 0.25% Triton-X100 in PBS). Primary antibody dilutions were prepared in 1% BSA (0.2% gelatin, 0.25% Triton-X100 in PBS) and incubated overnight (4 °C). After washing, the slides were incubated with the appropriate secondary antibody for 1 h at RT (1% BSA, 0.2% gelatin, 0.25% Triton-X100 in PBS) and counterstained with Hoechst-33258 for 15 min (1 µg/mL in PBS; 94403, Sigma-Aldrich). The slides were mounted using Anti-Fade Fluorescence Mounting Medium (AB104135, Abcam). The primary antibodies used for this co-localization experiment were rabbit-anti-PAC1 receptor (1:250, GeneTex Cat# GTX30026, RRID:AB_3097721), mouse-anti-GFAP (1:250, Cell Signaling Technology, Notting Hill, VIC, Australia, Cat# 3670, RRID:AB_561049), goat-anti-Olig2 (1:250, R&D Systems Cat# AF2418, RRID:AB_2157554), Goat anti-rabbit IgG (H+L), F(ab’)2 Fragment (Alexa Fluor^®^ 488 Conjugate) (1:500, Cell Signaling Technology, Notting Hill, VIC, Australia, Cat# 4412 (also 4412S), RRID:AB_1904025), Goat anti-mouse IgG H&L (Alexa Fluor^®^ 594) (1:500, Abcam Cat# ab150116, RRID:AB_2650601), Donkey Anti-Rabbit IgG H&L (Alexa Fluor 647; 1:500, (Abcam Cat# ab150063, RRID:AB_2687541) and Donkey Anti-Goat (Alexa Fluor 488; 1:500, Thermo Fisher Scientific Cat# A-11055, RRID:AB_2534102). All sections were imaged using the Zeiss Axioscan Z1 (20×, Zeiss, Macquarie Park, NSW, Australia). Figures were generated using OMERO.figure (v4.2.0).

### 4.6. Statistical Analysis

All data were analyzed and graphs generated using GraphPad Prism (v9.3.1). For pairwise comparisons (i.e., non-MS vs. MS), statistical significance was determined using the unpaired *t*-test. For comparisons involving three or more groups, statistics were computed using a one-way analysis of variance (ANOVA) followed by Sidak’s post hoc tests. *p*-values < 0.05 were considered statistically significant.

## Figures and Tables

**Table 1:** Demographic information of non-MS and MS donors. PMI = post-mortem interval. Adapted from [57].

Group	Age (Years)	Place of Birth	Sex	PMI (Hours)	MS Duration (Years)	Lesion Type
Control	79	Australia	Female	59	N/A	N/A
Control	82	England	Female	25	N/A	N/A
Control	83	Australia	Male	27	N/A	N/A
Control	73	Australia	Male	22	N/A	N/A
Control	73	Australia	Female	26.5	N/A	N/A
RRMS	70	Australia	Male	21	43	Chronic active
RRMS	80	Australia	Male	14	21.3	Chronic active
RRMS	40	Australia	Male	5	8	Chronic active
RRMS	72	Australia	Female	31	20	Chronic active
RRMS	79	New Zealand	Female	24	29.5	Chronic active
RRMS	82	Australia	Female	19	33.1	Chronic active—minimal regeneration
SPMS	57	Australia	Female	26.8	17.9	Chronic active—minimal regeneration
SPMS	68	Australia	Female	15	33.5	Chronic active—minimal regeneration
SPMS	69	New Zealand	Female	8.5	38	Chronic active—minimal regeneration
SPMS	84	Australia	Female	15	42	Chronic active—minimal regeneration
SPMS	47	Australia	Female	20.8	25.8	Chronic active—minimal regeneration
SPMS	55	Australia	Male	7	40.1	Chronic active—minimal regeneration
PPMS	36	Australia	Female	24	13	Chronic active
PPMS	83	Australia	Female	16	16	Chronic active
PPMS	73	Australia	Male	25	15.6	Chronic active—moderate regeneration
PPMS	73	England	Male	24	41	Chronic active—minimal regeneration

**Table 2:** Overview of the genes tested using RT-qPCR.

Accession Number	Gene	Primer Sequences (5′-3′)	Product Size (bp)
NM_001099733.2	Pituitary adenylate-cyclase-activating peptide (PACAP; *ADCYAP1)*	TAACGAGGCCTACCGCAAAG GTGAAGATCCCGTCCGAGTG	150
NM_003381.4	Vasoactive intestinal peptide (*VIP*)	AATAAGGCCCAGCTCCTTGTG TGTCACCCAACCTGAGAGCA	106
NM_001199635.2	Pituitary adenylate-cyclase-activating peptide receptor 1 (PAC1; *ADCYAP1R1*)	TTGGCATTATCGTCATCCTTGT AATGGTGGACAGTTCTGACATC	152
NM_004624.4	Vasoactive intestinal peptide receptor 1 (VPAC1; *VIPR1*)	TAAGCCTGAAGTGAAGATGGTC CATTGAGGAAGCAGTAGAGGAT	86
NM_003382.5	Vasoactive intestinal peptide receptor 2 (VPAC2; *VIPR2*)	CTCGCCCCCGTGAACAG GCACGTGATGTTGTCCCAGA	141
NM_022551.3	Ribosomal protein S18 (RP*S18*)	GAGGATGAGGTGGAACGTGT GGACCTGGCTGTATTTTCCA	115

## Data Availability

Raw data generated for this study can be made available upon reasonable request to authors.

## Supplementary Materials

The following supporting information can be downloaded at: https://www.mdpi.com/article/10.3390/ijms25168850/s1.
