# Supplementary material for: Differential Expression of PACAP/VIP Receptors in the Post-Mortem CNS White Matter of Multiple Sclerosis Donors
Source: Int J Mol Sci. 2024 Aug 14;25(16):8850. doi: 10.3390/ijms25168850 (PMC11354662; doi:10.3390/ijms25168850)
Supplement: Supplementary file 1 [file ijms-25-08850-s001.zip › ijms-3044089-supplementary.pdf]

Differential expression of PACAP/VIP receptors in the *post-mortem* CNS white matter of multiple sclerosis donors

Margo I. Jansen [1], Giuseppe Musumeci [2], Alessandro Castorina [1,*]

[1] Laboratory of Cellular & Molecular Neuroscience (LCMN), School of Life Sciences, Faculty of Science, University of Technology Sydney, P.O. Box 123, Sydney, NSW 2007, Australia; margo.jansen@student.uts.edu.au

[2] Department of Biomedical and Biotechnological Sciences, Human Anatomy and Histology Section, School of Medicine, University of Catania, Catania 95123, Italy. g.musumeci@unict.it.

* Correspondence: Alessandro.castorina@uts.edu.au

Supplementary Figure S1

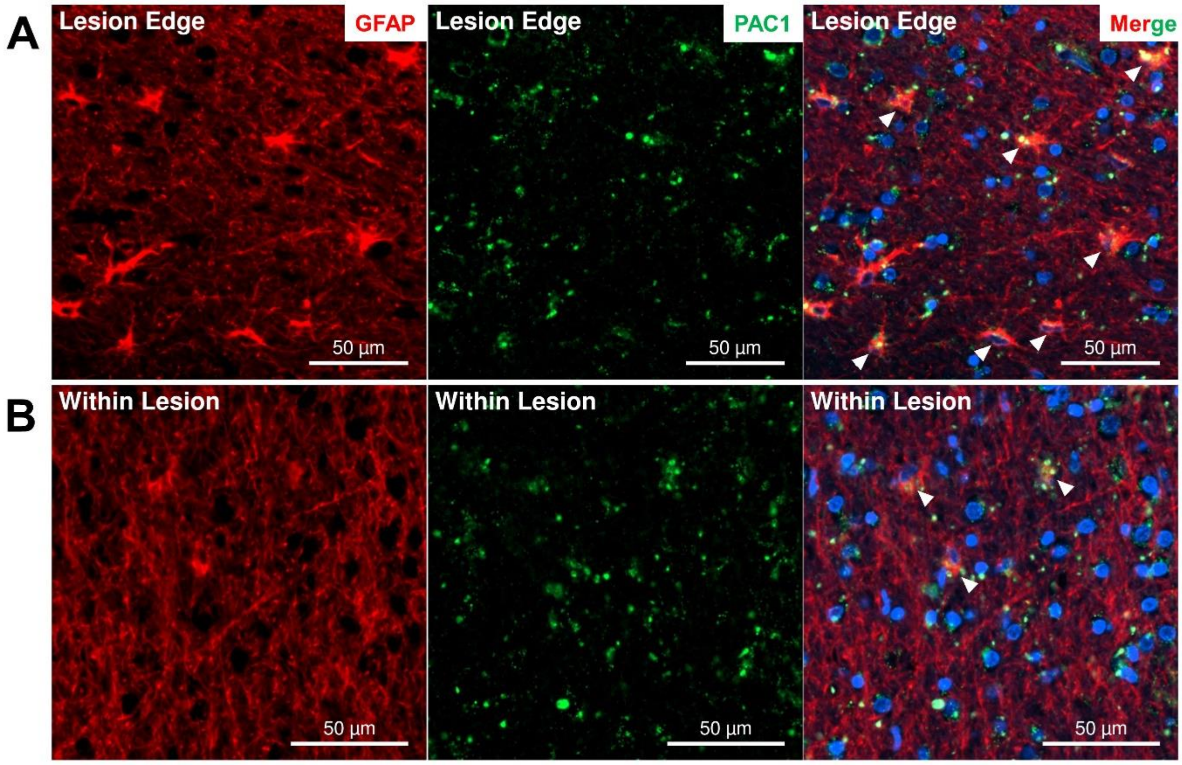

Figure S1. PAC1 colocalize to astrocytes in representative white matter lesions from a selected RRMS case. (A, top panels) Co-immunofluorescence demonstrating colocalization of PAC1 (green) in GFAP^{+} cells (red) along the lesion edges of an exemplary RRMS case. (B, bottom panels) Within the lesion centre of the same clinical case, PAC1^{+} astrocytes are still present but less abundant. White arrowheads point to PAC1^{+} astrocytes both in A and B. Scale bar = 50μm.
